# Di-n-butyl phthalate epigenetically induces reproductive toxicity via the PTEN/AKT pathway

**DOI:** 10.1038/s41419-019-1547-8

**Published:** 2019-04-05

**Authors:** Ran Li, Qian-wei Xing, Xiao-lu Wu, Lei Zhang, Min Tang, Jing-yuan Tang, Jing-zi Wang, Peng Han, Shang-qian Wang, Wei Wang, Wei Zhang, Guo-ping Zhou, Zhi-qiang Qin

**Affiliations:** 1 Department of Urology, The First Affiliated Hospital of Nanjing Medical University, 210029 Nanjing, China; 2 Department of Urology, Affiliated Hospital of Nantong University, 226001 Nantong, China; 3 Department of Pediatrics, The First Affiliated Hospital of Nanjing Medical University, 210029 Nanjing, China; 4 Jiangsu Province Hospital of Chinese Medicine, Affiliated Hospital of Nanjing University of Chinese Medicine, 210029 Nanjing, China; 5 Department of Urology, Nanjing First Hospital, Nanjing Medical University, 210006 Nanjing, China

## Abstract

Di-n-butyl phthalate (DBP) is a kind of ubiquitous chemical linked to hormonal disruptions that affects male reproductive system. However, the mechanism of DBP-induced germ cells toxicity remains unclear. Here, we demonstrate that DBP induces reduction of proliferation, increase of apoptosis and DNA damage dependent on the PTEN/AKT pathway. Mechanistically, DBP decreases PTEN promoter methylation and increases its transcriptional activity, leading to increased PTEN expression. Notably, DNMT3b is confirmed as a target of miR-29b and miR-29b-mediated status of PTEN methylation is involved in the effects of DBP treatment. Meanwhile, DBP decreases AKT pathway expression via increasing PTEN expression. In addition, the fact that DBP decreases the sperm number and the percentage of motile and progressive sperm is associated with downregulated AKT pathway and sperm flagellum-related genes. Collectively, these findings indicate that DBP induces aberrant PTEN demethylation, leading to inhibition of the AKT pathway, which contributes to the reproductive toxicity.

## Introduction

Exposure to endocrine-disrupting chemicals (EDCs) is an important environmental factor that may contribute to male congenital malformation and infertility, including cryptorchidism, hypospadias, low sperm count, and testicular cancer^1^. Di-n-butyl phthalate (DBP), a well-known EDC, is widely used in industrial productions. The use or disposal of plastics leads to ubiquitous exposure to DBP, which adversely affects male reproductive health^2^.

Recently, a series of studies on DBP-induced male reproductive toxicity have been performed, which revealed many important underlying mechanisms about the effects of DBP on male reproductive toxicity. Sertoli cells are essential for spermatogenic cell survival via creating a microenvironment. Wang et al. reported PI3K/AKT/mTOR signaling pathway was associated with DBP-induced apoptosis of testicular Sertoli cells in vitro^3^. Autophagy, a specifically intracellular self-defense process, was found in prepubertal rat testis germ cells after DBP-induced endoplasmic reticulum (ER) stress. DBP-induced ER stress and autophagy was considered having a cytoprotective role against apoptosis in vitro and in vivo^4^. In addition, growing evidence indicated that oxidative stress was an important factor to explain the DBP toxicity mechanism. DBP-induced seminiferous tubules atrophy and seminiferous epithelial cells disintegrated, at least partly, via activating oxidative stress in adult rat testes^5^. Our previous study indicated sulforaphane could alleviate DBP-induced testicular oxidative stress injury in male mice offsprings via activating Nrf2/ARE pathway^6^. However, the mechanism of DBP-induced germ cells toxicity remains to be elucidated.

Epigenetic modification has been increasingly recognized as an important potential biological mechanism through which exposures can induce adverse effects later in life. DNA methylation is the most commonly studied type of epigenetic modification that plays an important role in gene regulation and various cellular processes^7^. DNA methylation is mainly regulated by DNA methyltransferases (DNMTs), including DNMT1, DNMT3a, and DNMT3b. DNMT3a and DNMT3b preferentially catalyzed de novo methylation, and subsequently maintained by DNMT1 in a replication-dependent manner^8,9^. Previous studies have shown associations between phthalate exposure and epigenetic modifications. One study found that phthalate exposure in utero was related to DNA methylation status of a series of genes regulating spermatogenesis, antiandrogenic effect, cell proliferation, and protein secretion using cord blood samples^10^. Transgenerational differential DNA methylation regions in sperm epigenome were observed between plastic derived compounds-treated F3 generation rats and controls^11^. In addition, evidence from 562 Chinese adult men shown that 5mdC and 5hmdC were associated with phthalate exposure and semen quality^12^.

miRNAs, with the range of ~20 nucleotides, are endogenously expressed and regulate gene expression on the posttranscriptional level in many organisms^13^. Recent studies indicate that microRNA (miRNA) plays an important role in genetic controlling of differentiation and pluripotency of germ cells^14^. Some studies have indicated miRNAs could regulate DNA methylation by targeting DNMTs. Adiponectin inhibits hepatic stellate cell activation via regulating PTEN expression. Mechanistically, upregulation of miR-29b induced by adiponectin can suppress DNMT3b transcription, leading to reduced PTEN methylation and ultimately suppressing the PI3K/AKT pathway^15^. HOTAIR, as a long intergenic non-coding RNA, downregulates miR-29b expression, leading to enhanced DNA methylation of PTEN promoter, which induces decreased PTEN expression^16^. In this study, we asked whether miR-29b regulate PTEN methylation in germ cells.

PTEN is a well-known tumor suppressor gene, which depends largely on its lipid phosphatase activity. As such, PTEN is also a key factor involved in proliferation, survival, energy metabolism, and cellular architecture^17^. As a key AKT pathway inhibitor, PTEN expression is frequently regulated by aberrant DNA methylation^18^. In addition, Telomerase reverse transcriptase inhibition leads to the PTEN promoter demethylation and increased PTEN expression in hepatocellular carcinoma cells^18^. AKT (protein kinase B), which is a serine-threonine kinase that activates key multifunctional downstream targets such as glycogen synthase kinase 3, Forkhead Box O), and mechanistic target of rapamycin (mTOR). Both AKT and mitogen-activated protein kinase pathways are indispensable for spermatogonial stem cell (SSC) self-renewal^19^. Previous study has revealed that PTEN/AKT pathway might be associated with DBP-induced apoptosis of testicular Sertoli cells in vitro^3^. However, how DBP modulates PTEN/AKT pathway and whether DNA methylation is involved in it has not yet been fully described. Here, we hypothesized that DBP might regulate PTEN expression via either DNMTs, miR-29b, or both. Hence this study aimed to determine whether DBP induces reproductive toxicity via PTEN/AKT pathway and investigate the molecular mechanisms of DBP-mediated increased PTEN expression.

## Results

### DBP induces apoptosis, proliferation inhibition, and DNA damage in germ cells

**Fig. 1:**
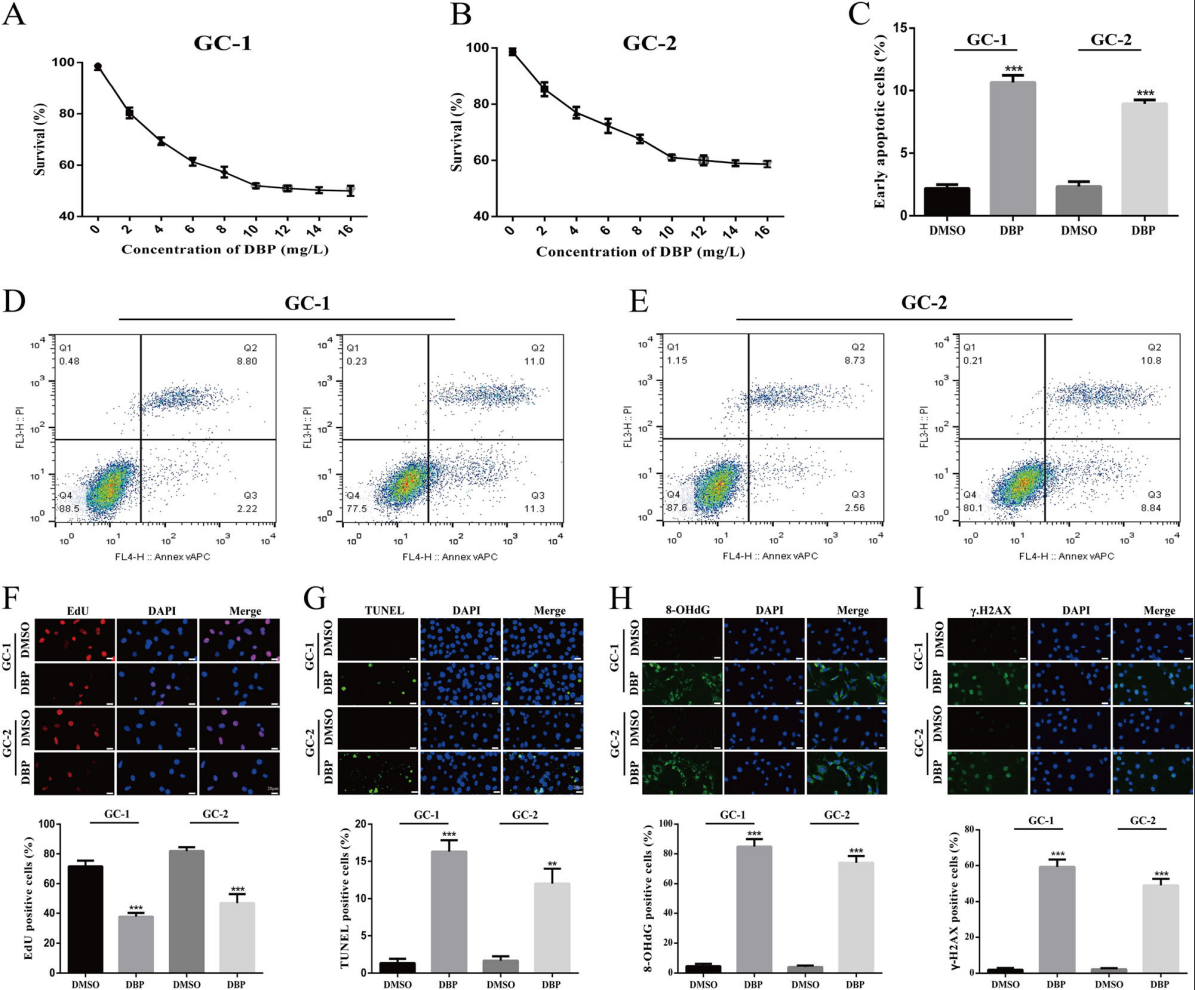
DBP induces apoptosis, proliferation inhibition and DNA damage in germs. **a**, **b** Cell viability was detected by CCK-8 proliferation assay in GC-1 (**a**) and GC-2 (**b**) cells after treated with different concentrations of DBP. **c**–**e** Early apoptotic percentage was determined by flow cytometry in GC-1 (**d**) and GC-2 (**e**) cells after treated with DBP. **f**–**i** Representative immunofluorescence (IF) images showing the number of EdU- (**f**), TUNEL- (**g**), 8-OHdG- (**h**), and γ-H2AX (**i**) -positive cells in GC-1 and GC-2 cells after treated with DBP. Scale bar, 20 μm. All measurements are shown as the means ± SD from three independent experiments, **p < 0.01, ****p < 0.001

We treated GC-1 and GC-2 cells with different concentrations of DBP for 24 h and then assessed cell viability using CCK-8 proliferation assay. From Fig. 1a, b, IC50 values of DBP we calculated were 11.495 and 22.166 mg/L in GC-1 and GC-2 cells, respectively. In consideration of the dose of DBP is relatively high in animal models^6,20^, the dose of 10 mg/L was chosen for subsequent studies in germ cells. We detected the frequency of proliferating and apoptotic cells using EdU incorporation assay, TUNEL assay, and Annexin-V-APC/PI dual staining assay. The results indicated that DBP significantly decreased the percentage of EDU positive cells (Fig. 1f), whereas increased the percentage of TUNEL positive cells (Fig. 1g). In addition, flow cytometry analysis showed that DBP significantly induced early apoptosis in both GC-1 and GC-2 cells (Fig. 1d, e). In a state of oxidative stress, excess reactive species can damage various cellular components, including membrane lipids and DNA. The response of germ cells to DNA damage include initiating DNA-PK and ATM/ATR to activate DNA repair, cell cycle arrest, and/or apoptosis^21,22^. Hence, we hypothesized that DNA damage could generate in GC-1 and GC-2 cells after treated with DBP. Immunofluorescence (IF) staining was used to examine 8-OHdG and γ-H2AX levels. Upon DBP treatment, increased 8-OHdG and γ-H2AX levels were observed in DBP-treated cells compared with controls (Fig. 1h, i). These data indicate DBP can induce reduction of proliferation, increase of apoptosis, and DNA damage in germ cells.

### AKT pathway is downregulated in DBP-treated germ cells

**Fig. 2:**
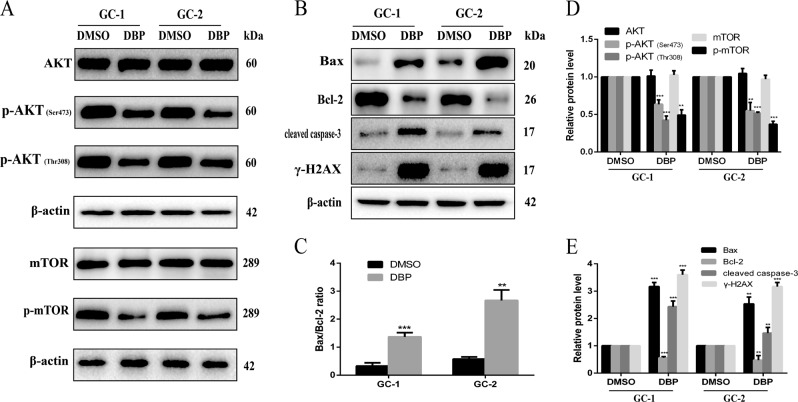
AKT pathway is downregulated in DBP-treated germ cells. **a** Representative WB images showing the protein levels of AKT, p-AKT (Ser473), p-AKT (Thr308), mTOR, p-mTOR in GC-1, and GC-2 cells after treated with DBP. Protein loading is indicated by β-actin. **b** Representative WB images showing the protein levels of Bax, Bcl-2, cleaved caspase-3, and γ-H2AX in GC-1 and GC-2 cells after treated with DBP. Protein loading is indicated by β-actin. **c** Quantitative analysis of Bax/Bcl-2 ratio for Fig. 3b. **d** Quantitative analysis for Fig. 3a. **e** Quantitative analysis for Fig. 3b. All measurements are shown as the means ± SD from three independent experiments, ***p < 0.01, ****p < 0.001

Previous studies showed DBP increased PTEN expression and decreased AKT activity in Sertoli cells^3^. Hence, we determined whether this scenario also occurred in GC-1 and GC-2 cells. We examined AKT/mTOR expression in DBP-treated cells and controls. The protein expression of p-AKT (Ser473), p-AKT (Thr308), and p-mTOR was significantly decreased, whereas AKT and mTOR expression were not significantly different in DBP-treated cells compared with controls (Fig. 2a). Due to the fact that AKT is also an anti-apoptotic protein via regulating several apoptosis-related factors, including a pro-apoptotic protein, Bax, and an anti-apoptotic protein, Bcl-2^23^, we explored the expression of Bcl family core members Bcl-2 and Bax in GC-1 and GC-2 cells. The western blot (WB) results indicated DBP increased Bax expression and decreased Bcl-2 expression in GC-1 and GC-2 cells (Fig. 2b). And there was an approximate 4.7-fold and 4-fold increase in the Bax/Bcl-2 ratio, respectively, in GC-1 and GC-2 cells after exposure to DBP compared with controls (Fig. 2c). In addition, cleaved caspase-3, a marker for apoptosis, was also increased in DBP-treated group (Fig. 2b).

### DBP induces cytotoxicity dependent on inhibiting AKT pathway

SC79, a novel AKT-specific activator, is confirmed capable of inducing AKT phosphorylation at both Ser473 and Thr308 sites^24^. Next, the effects of SC79 on DBP-induced cytotoxicity in germ cells were evaluated in the pretreatment of cells with increasing concentrations of SC79 for 2 h before the addition of DBP in the concentration of 10 mg/L and then we assessed cell viability 24 h later. Indeed, SC79 increased cell survival in a dose dependent manner up to 5 μg/mL in both GC-1 and GC-2 cells (Fig. S1A and S1B), which was chosen for subsequent studies.

**Fig. 3:**
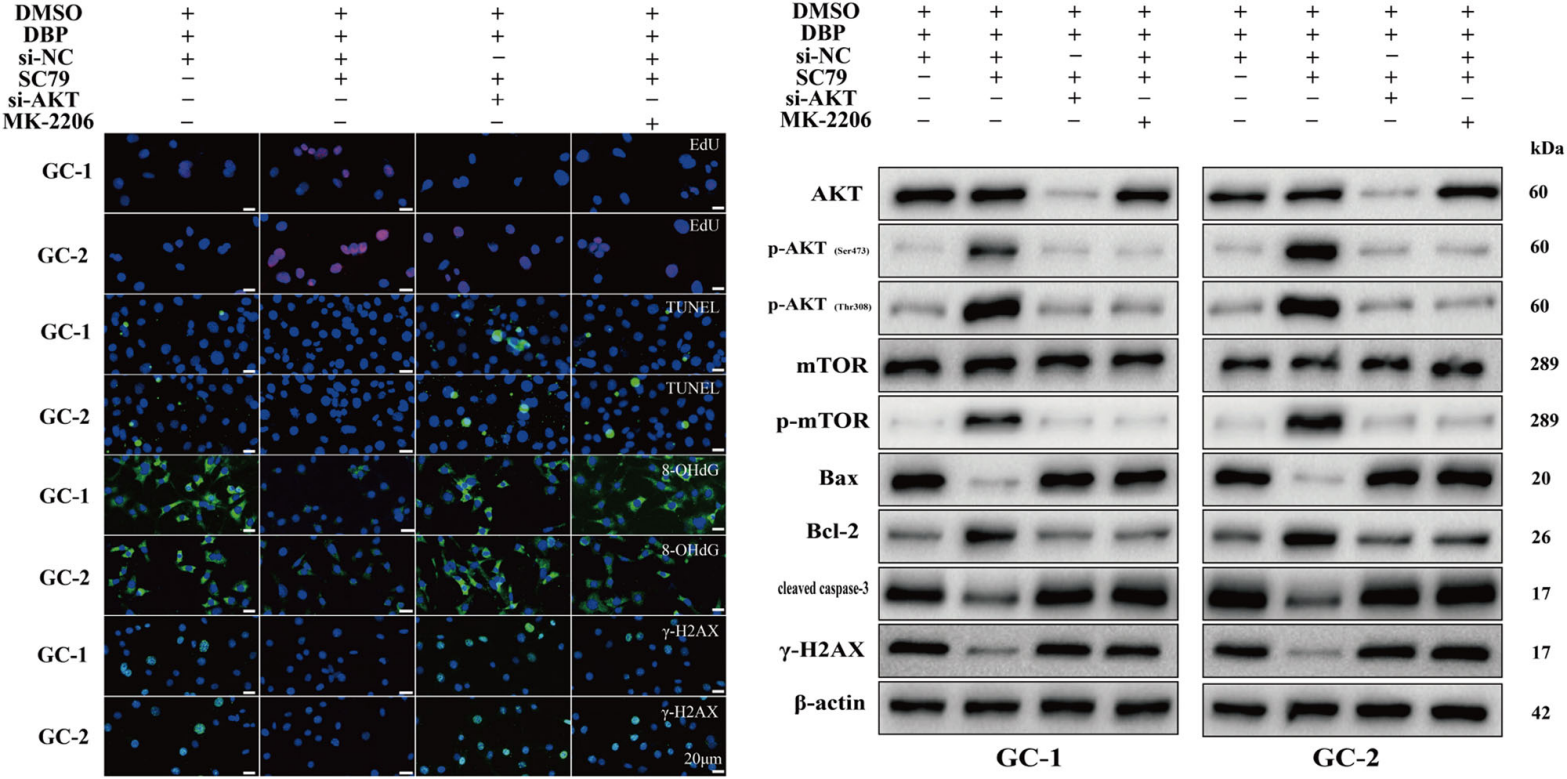
DBP induces cytotoxicity dependent on inhibiting AKT pathway. **a** Representative IF images showing the number of EdU, TUNEL, 8-OHdG, and γ-H2AX positive cells in GC-1 and GC-2 cells after DBP treatment, SC79 treatment, AKT knockdown, or MK-2206 treatment. Scale bar, 20 μm. **b** Representative WB images showing the protein levels of AKT, p-AKT (Ser473), p-AKT (Thr308), mTOR, p-mTOR, Bax, Bcl-2, cleaved caspase-3, and γ-H2AX in GC-1 and GC-2 cells after DBP treatment, SC79 treatment, AKT knockdown, or MK-2206 treatment. Protein loading is indicated by β-actin. All measurements are shown as the means ± SD from three independent experiments

Either genetic knockdown of AKT (Fig. S1C and S1D) or pharmacological inhibition of AKT phosphorylation (Fig. S1F) was applied to diminish hyperphosphorylation of AKT induced by SC79 (Fig. S1E). Expectedly, AKT knockdown or inhibition of AKT phosphorylation abolished the cytoprotective effect of SC79. In the presence of SC79 and DBP, CCK-8 assay indicated AKT knockdown or inhibition of AKT phosphorylation decreased the cell viability compared with controls. (Fig. S1G and S1H). In addition, SC79 pretreatment significantly increased the percentage of proliferative cells and decreased the percentage of apoptotic and DNA damage cells exposed to DBP (Fig. 3a). Meanwhile, WB results indicated SC79 pretreatment restored AKT pathway expression compared with DBP-treated groups (Fig. 3b). However, upon AKT knockdown or inhibition of AKT phosphorylation, the protective role of SC79 on cell survival and anti-DNA damage was fully reversible (Fig. 3a) and the effect of SC79 on the AKT pathway was also blocked (Fig. 3b). These results indicate AKT pathway plays an important role in DBP-induced cytotoxicity and SC79 may be a prospective reagent to protect against DBP-induced adverse effects in germ cells.

### DBP-mediated AKT activity by regulating PTEN expression in germ cells

**Fig. 4:**
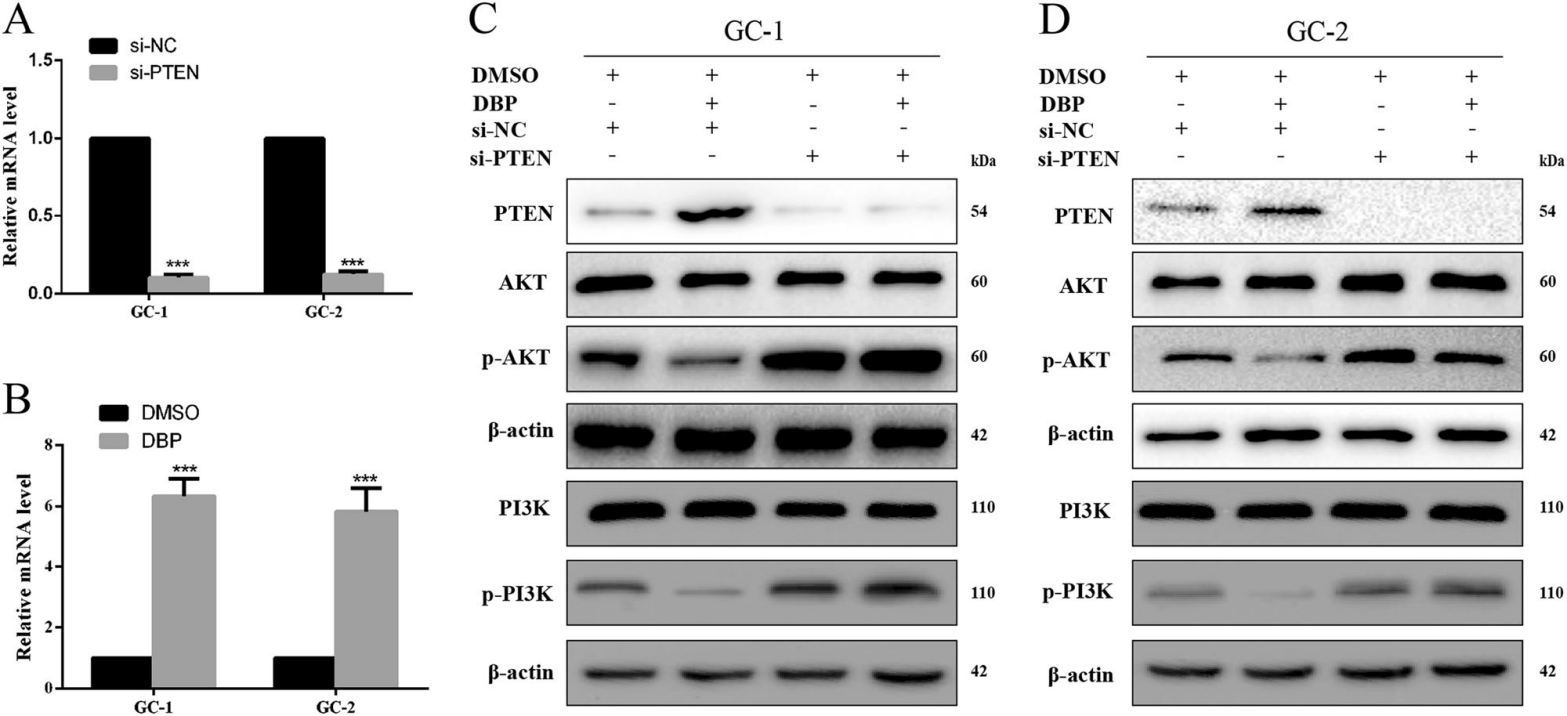
DBP-mediated AKT activity by regulating PTEN expression in germ cells. **a** qRT-PCR was used to detect the efficiency of PTEN knockdown in GC-1 and GC-2 cells. **b** qRT-PCR was used to detect PTEN expression after treated with DBP in GC-1 and GC-2 cells. **c**, **d** GC-1 (**c**) and GC-2 (**d**) cells were transfected with PTEN siRNA or treated with DBP alone, and together, respectively. And then samples were analyzed for PTEN, AKT, p-AKT, PI3K, and p-PI3K expression. All measurements are shown as the means ± SD from three independent experiments, ****p < 0.001

A diminished AKT pathway abundance was observed in DBP-treated cells, which was coupled with increased PTEN expression (Fig. 4c, d). qRT-PCR showed that DBP increased PTEN expression in GC-1 and GC-2 cells (Fig. 4b). A blockade of PTEN in GC-1 and GC-2 cells using PTEN specific siRNA (Fig. 4a) restored the p-PI3K and p-AKT expression (Fig. 4c, d), which suggests that PTEN is involved in DBP-mediated AKT/PI3K activity in germ cells.

### DBP induces PTEN promoter demethylation and increases its promoter transcriptional activity

**Fig. 5:**
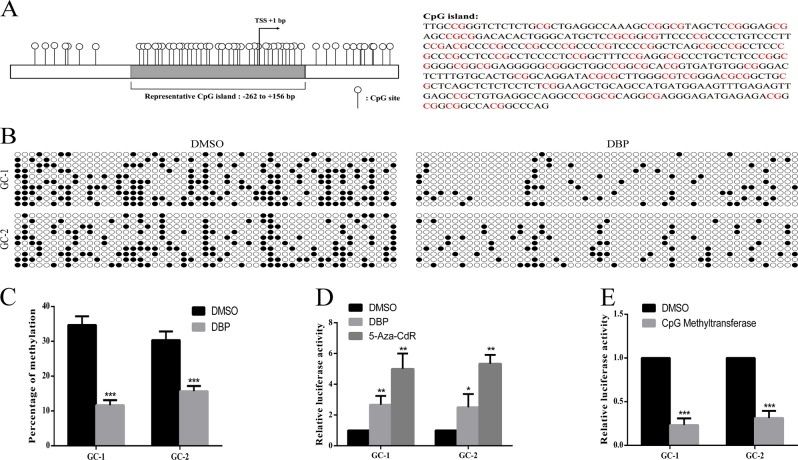
DBP induces PTEN promoter demethylation and global decline in DNA methylation. **a** The schematic diagram and sequence of representative CpG Island of the PTEN promoter region. **b** Representative bisulfite-sequencing PCR results for the CpG island in the PTEN promoter region in GC-1 and GC-2 cells. Each row represents the result of a clone sequence, each column represents a CpG site of the CpG island (10 clones, 53 CpG sites). The solid spots represent methylated CpG sites, the hollow spots represent unmethylated CpG sites. **c**–**e** Percentage of methylation for the CpG island above in GC-1 and GC-2 cells in the treatment of DBP, 5-Aza-CdR, or CpG Methyltransferase. All measurements are shown as the means ± SD from three independent experiments, **p < 0.05, ***p < 0.01, ****p < 0.001

Given the observations above, we sought to determine whether DBP regulates PTEN expression via affecting its promoter methylation. We detected the methylation level at 53 cytosine-phosphate-guanine (CpG) sites within the CpG island containing the transcriptional start site in the PTEN locus by bisulfite-sequencing analysis (Fig. 5a). Compared with the controls, significant decrease in PTEN promoter methylation was observed in DBP-treated germ cells (Fig. 5b). Generally, DNA methylation regulates gene expression via changing its promoter transcriptional activity. To determine whether DBP-induced hypomethylation in PTEN promoter led to its transcriptional activity changes, we detected PTEN promoter transcriptional activity using luciferase reporter assays.

We inserted PTEN genomic DNA fragment −262/+156 containing the CpG island in the PTEN promoter region into the pGL3-Basic vector named as pGL-262/+156 and then transfected the plasmid into GC-1 and GC-2 cells. Recombinant luciferase reporter construct was methylated in vitro using CpG Methyltransferase. GC-1 and GC-2 cells were incubated with 5-Aza-CdR for 48 h to block methylation. As shown in Fig. 5d, DBP induced a marked increase in PTEN promoter transcriptional activity in germ cells compared with controls. In addition, 5-Aza-CdR also increased the PTEN promoter transcriptional activity, whereas CpG Methyltransferase decreased its promoter transcriptional activity (Fig. 5d, e).

### miR-29b and DNMT3b are required for DBP-induced PTEN demethylation

**Fig. 6:**
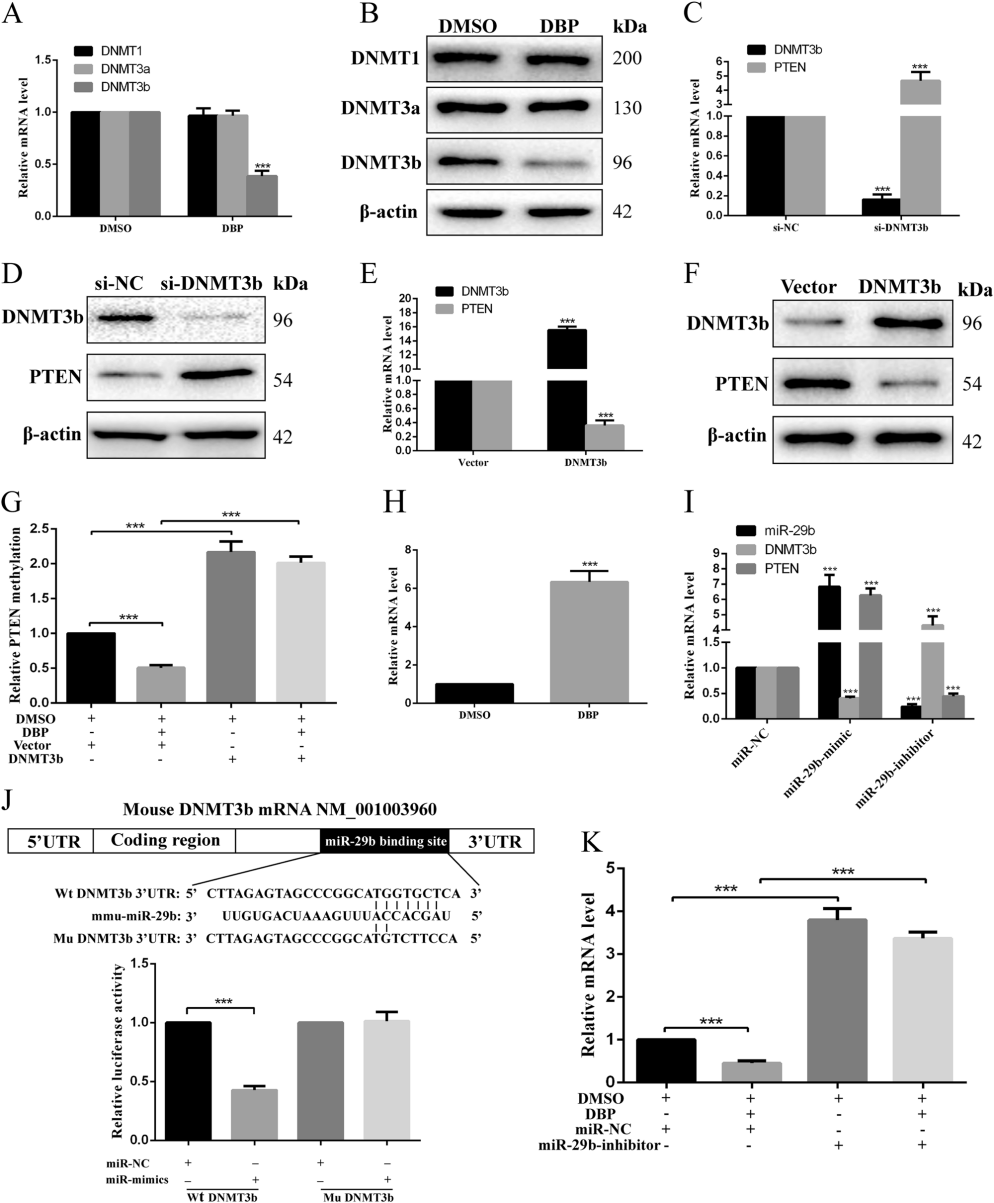
miR-29b and DNMT3b are required for DBP-induced PTEN demethylation. **a** DNMT1, DNMT3a and DNMT3b were detected using qRT-PCR in GC-2 cells after treated with DBP. **b** Representative WB images showing the expression of DNMT1, DNMT3a, and DNMT3b in GC-2 cells after treated with DBP. **c**, **d** qRT-PCR (**c**) and WB (**d**) results showed the expression of DNMT3b and PTEN in the treatment of si-NC or si-DNMT3b in GC-2 cells. **e**, **f** qRT-PCR (**e**) and WB (**f**) results showed the expression of DNMT3b and PTEN in GC-2 cells after DNMT3b overexpression. **g** Percentage of methylation for the CpG island of PTEN in GC-2 cells in the treatment of DMSO, DBP, vector, or DNMT3b overexpression. **h** miR-29b expression was detected by qRT-PCR in GC-2 cells after treated with DBP. **i** qRT-PCR showed the expression of miR-29b, DNMT3b, and PTEN in the treatment of miR-NC, miR-29b-mimic, or miR-29b-inhibitor in GC-2 cells. **j** The position of the binding sites was numbered relative to the first nucleotide of the 3′-UTR. Mutations were introduced into DNMT3b 3′-UTR that matched the seed region of miR-29b as shown in DNMT3b Mu. Luciferase activity was detected using dual-luciferase assay in GC-2 cells co-transfected with luciferase constructs containing the DNMT3b Wt or Mu 3′-UTR and miR-29b mimics or scrambled oligonucleotides as the negative control. **k** qRT-PCR showed the expression of DNMT3b in the treatment of DMSO, DBP, miR-NC, or miR-29b-inhibitor. All measurements are shown as the means ± SD from three independent experiments, ****p < 0.001

Generally, DNMTs, including DNMT1, DNMT3a, and DNMT3b, are essential in the regulation of global DNA methylation patterns. To determine whether DBP induced PTEN demethylation via DNMTs, expression of DNMTs were detected in GC-2 cells. The results indicated DBP decreased DNMT3b expression, whereas DNMT1 and DNMT3a expression were not found significant difference between DBP-treated group and control (Fig. 6a, b). To understand whether DNMT3b is involved in regulating PTEN expression, its expression was measured after ectopic DNMT3b expression or knockdown. We found DNMT3b knockdown increased PTEN expression (Fig. 6c, d), whereas DNMT3b overexpression led to a decrease of PTEN expression (Fig. 6e, f). Importantly, Overexpression of DNMT3b downregulation in DBP-treated cells abolished DBP-induced PTEN demethylation (Fig. 6g), suggesting DNMT3b is required for DBP-induced PTEN demethylation.

Recent studies showed that miR-29b could increase PTEN methylation via decreasing DNMT3b expression^15,16^. Next, we determined whether miR-29b was involved in regulating DNMT3b expression. In GC-2 cells, DBP increased miR-29b expression (Fig. 6h). Interestingly, RNA sequencing results revealed that miR-29b expression was increased in DBP-treated group (Fig S2A). Next, we built a miRNA-Gene-Network and found miR-29b in the upregulated miRNAs had the most targets (degree 94) (Fig S2B). Degree means the contribution one miRNA to the genes around or the contribution one gene to the miRNAs around. The key miRNAs and genes in the network always have the highest degrees. In addition, miR-29b regulated some important targets, including DNMT3b, Ddx39a, Wnt16, Ebf1 and so on, which are known to regulate apoptosis, proliferation, DNA methylation, and spermatogenesis, suggesting that miR-29b may play an important role in DBP-induced toxicity. To understand the role of miR-29b in regulating DNMT3b and PTEN expression, miR-29b mimic and inhibitor were used to overexpress or knock down miR-29b. As shown in Fig. 6i, miR-29b overexpression decreased DNMT3b expression and increased PTEN expression, whereas miR-29b knockdown increased DNMT3b expression and decreased PTEN expression. Using bioinformatic analysis (Targetscan), DNMT3b is predicted as a potential target of miR-29b, and we generated a DNMT3b 3′-UTR luciferase reporter containing the miR-29b-binding sites (Wt DNMT3b) or mutated sites (Mu DNMT3b). As shown in Fig. 6j, miR-29b overexpression decreased DNMT3b luciferase activity in Wt DNMT3b group, whereas no significant differences were observed after miR-29b overexpression in Mu DNMT3b group. These data indicated that DNMT3b was a target of miR-29b. To determine whether miR-29b was involved in DBP-induced DNMT3b decrease, we knocked down miR-29b expression in DBP-treated cells. The results indicated miR-29b knockdown abolished DBP-induced DNMT3b decrease (Fig. 6k).

### DBP induces reproductive toxicity via AKT pathway and PTEN methylation might be involved in it in male mice offsprings

**Fig. 7:**
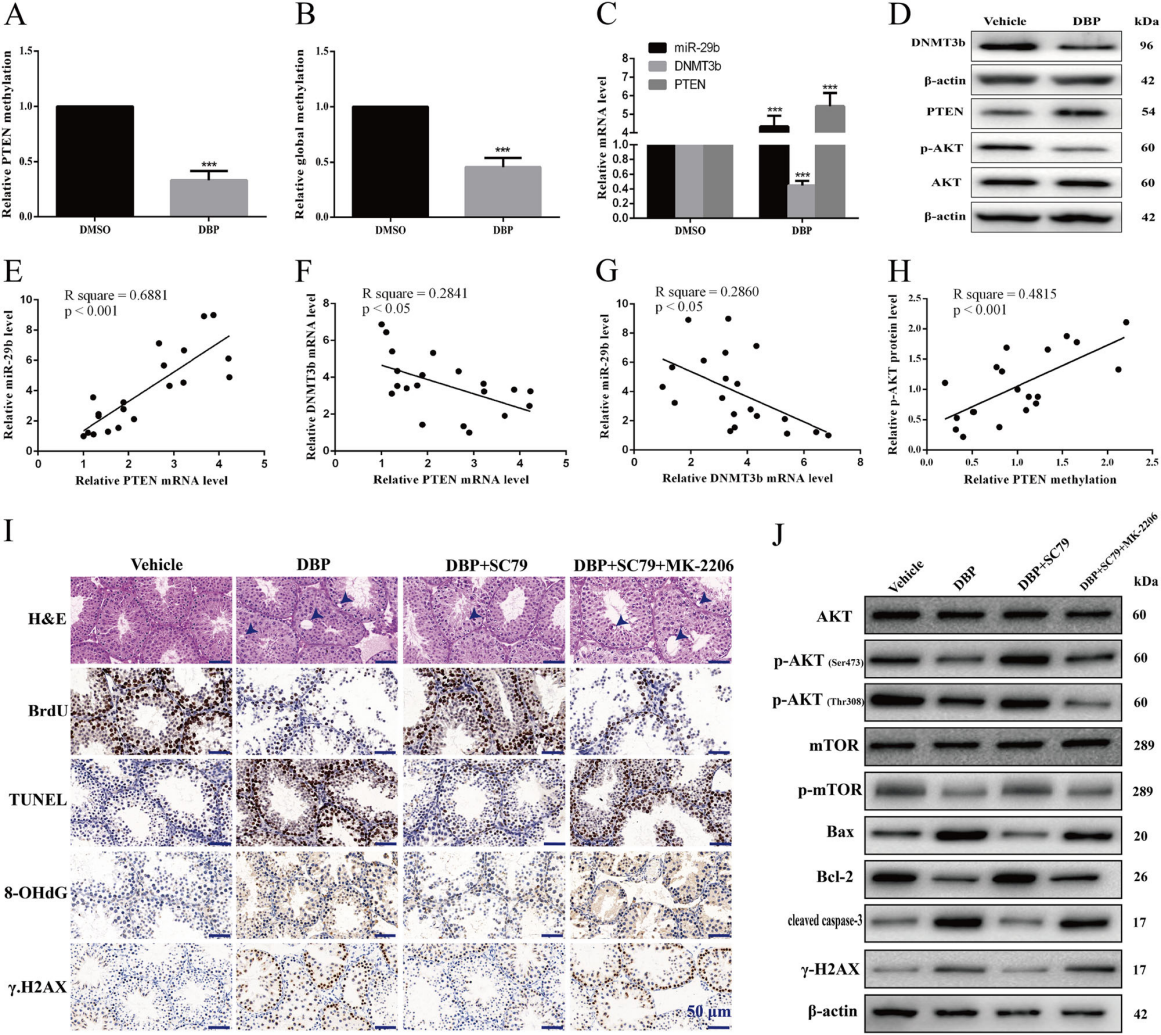
DBP induces reproductive toxicity via AKT pathway and PTEN methylation might be involved in it in male mice offsprings. **a** Bisulfite sequencing PCR results for the CpG island in the PTEN promoter region in DBP administration male mice offsprings. **b** Quantitative analysis for the results of global methylation in DBP administration group. **c** qRT-PCR was used to detect miR-29b, DNMT3b, and PTEN levels in DBP administration group. **d** Representative WB images showing the protein levels of DNMT3b, PTEN and p-AKT in DBP administration group. **e**–**h** Pearson correlation test results showing the correlation between PTEN mRNA level and miR-29b level (**e**) and between PTEN mRNA level and DNMT3b mRNA level (**f**) and between DNMT3b mRNA level and miR-29b level (**g**) and between PTEN methylation and p-AKT protein abundances (**h**). **i** Representative hematoxylin and eosin (h&e) staining showed the testicular morphology after DBP treatment, SC79 treatment, or MK-2206 treatment. Representative immunohistochemistry (IHC) images showing BrdU, TUNEL, 8-OHdG, and γ-H2AX positive areas in testicular tissues after DBP treatment, SC79 treatment or MK-2206 treatment. Scale bar, 50 μm. **j** Representative WB images showing the protein levels of AKT, p-AKT (Ser473), p-AKT (Thr308), mTOR, p-mTOR (S2448), Bax, Bcl-2, cleaved caspase-3, γ-H2AX in GC-1 and GC-2 cells after DBP treatment, SC79 treatment, or MK-2206 treatment. Protein loading is indicated by β-actin. All measurements are shown as the means ± SD from three independent experiments, ****p < 0.001

To confirm the findings in vitro, we used the pregnant mice as a research model^6^ to study the impact of exposure to DBP and the mechanisms involved in it. Firstly, we detected the methylation status of PTEN promoter in testicular tissues using bisulfite-sequencing PCR (BSP). The results showed there was a decrease in PTEN methylation in DBP administration group (Fig. 7a). In addition, we compared the global DNA methylation between control and DBP administration group using Luminometric Methylation Assay (LUMA), and observed significantly reduced global DNA methylation in DBP administration group compared with controls (Fig. 7b). Meanwhile, DBP increased miR-29b and PTEN expression, whereas decreased DNMT3b and p-AKT expression (Fig. 7c, d). Pearson correlation test indicated there was a significantly positive correlation between PTEN mRNA level and miR-29b level in testicular tissues (Fig. 7e). Negative correlations between PTEN mRNA level and DNMT3b mRNA level (Fig. 7f) and between DNMT3b mRNA level and miR-29b level (Fig. 7g) were observed. As shown in Fig. 7h, there was a significantly positive correlation between PTEN methylation and p-AKT protein abundance.

Next, we sought to determine whether AKT pathway involved in DBP-induced toxicity in vivo. Firstly, we evaluated the characteristics of testicular morphology. H&E staining showed that abnormal germ cells and missed cell layers (arrows) frequently existed in seminiferous tubules especially in the DBP and DBP+SC79+MK-2206 administration groups. In accordance with the results in GC-1 and GC-2 cells, Brdu positive cells were decreased significantly, whereas TUNEL, 8-OHdG, and γ-H2AX positive cells were increased significantly in DBP-treated groups compared with controls. SC79 attenuated the adverse effect above of DBP on germ cells and MK-2206 abolished SC79-mediated protective effects of germ cells (Fig. 7i). According to the WB results, p-AKT, p-mTOR, and Bcl-2 were decreased in DBP group compared with vehicle group, whereas Bax, cleaved caspase-3, and γ-H2AX were increased. In addition, p-AKT, p-mTOR, and Bcl-2 were increased in DBP+SC79 group compared with DBP group and SC79 significantly decreased Bax, cleaved caspase-3, and γ-H2AX expression level compared with DBP group. However, the effects of SC79 on AKT pathway were reversible with MK-2206 (Fig. 7j).

### DBP induces low sperm motility is associated with AKT pathway and sperm flagellum-related genes

**Fig. 8:**
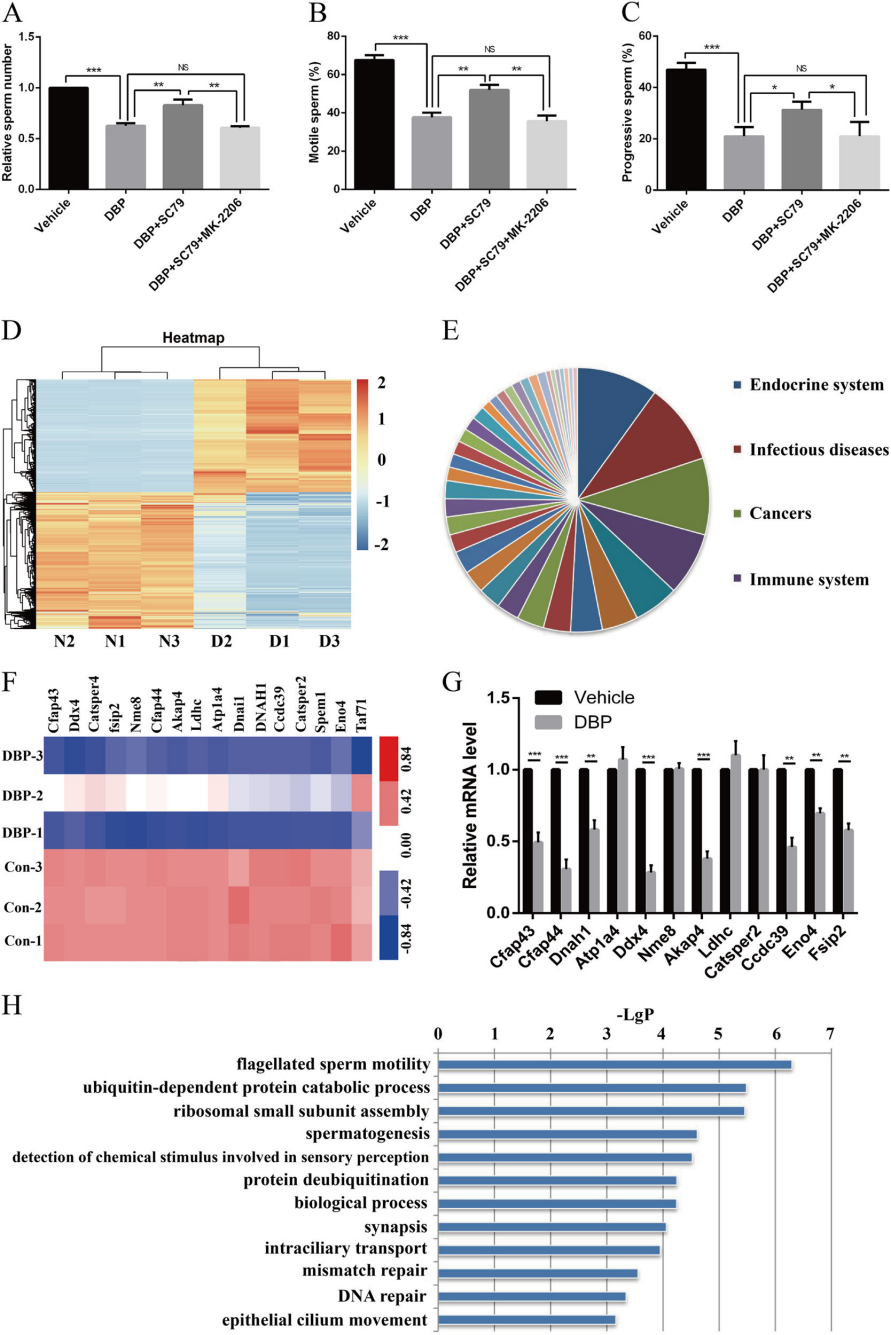
DBP induces low sperm motility and reduces sperm flagellum-related genes expression. **a**–**c** The number of sperms (**a**) and the percentages of motile sperm (**b**) and progressive sperm (**c**) in offsprings in the administration of DBP treatment, SC79 treatment, or MK-2206 treatment in the embryonic period. **d** Mean-centered, hierarchical clustering of all genes altered in DBP administration rats. **e** The classification of enriched pathway in the RNA sequencing results. **f** Mean-centered, hierarchical clustering of sperm flagellum-related genes in DBP administration rats. **g** Expression of sperm flagellum-related genes in DBP administration group were detected by qRT-PCR analysis. **h** Gene GO terms significantly enriched in the DBP stimulation gene subsets. All measurements are shown as the means ± SD from three independent experiments, **p < 0.05, ***p < 0.01, ****p < 0.001, NS, not significant

Since DBP could induce germ cells toxicity in vivo, we speculated sperm motility might also be influenced exposed to DBP. The results revealed DBP could decrease sperm numbers and the percentage of motile and progressive sperm. In addition, SC79 administration restored the adverse effects of DBP on sperms, which was reversible with MK-2206 (Fig. 8a–c). The RNA sequencing results in DBP administration rat model indicated DBP mainly influenced endocrine system, which was in accordance with the fact that DBP is a kind of EDCs (Fig. 8e). DBP remarkably reduced sperm flagellum-related genes expression including Cfap43, Cfap44, Dnah1 and so on (Fig. 8f, g). According to the RNA sequencing results, DBP stimulation regulated genes related to important signaling processes, including the flagellated sperm motility (Fig. 8h).

## Discussion

The testicular dysgenesis syndrome (TDS) hypothesis has become the main focus for studies into the origins of male reproductive disorders^25,26^. Although TDS might result from genetic mutations, recent evidence suggested that environmental exposures could be the most important factors contributing to the occurrence of TDS^26^. Animal experiments suggested DBP could induce a spectrum of TDS-related disorders^27–29^, and the adverse effects of DBP on germ cells were observed in a human fetal testis xenografts model^30^. This study focused on the mechanisms of DBP-induced reproductive toxicity.

Spermatogenesis is a dynamic homeostasis process rely on the balance between germ cell proliferation and apoptosis^31^. Growing evidence demonstrates that DBP is detected in human semen, which indicates that human spermatocytes or sperms could be damaged targets of DBP^32,33^. Hence, GC-1 and GC-2 cells were used as cell models to demonstrate whether DBP affected the balance between germ cell proliferation and apoptosis. Tubular atrophy is accompanied by spermatogenic cell apoptosis^34^. Several mechanisms have been proposed to explain it. For example, collapse of vimentin filaments of Sertoli cells lead to inadequate metabolic support for germ cells^35^. Zhang et al. reported ER stress and autophagy were involved in DBP-induced germ cells apoptosis^4^. We found that DBP induced reduction of proliferation, increase of apoptosis via inhibiting PTEN/AKT pathway and DNA damage might be involved in it. The oxidative damage to DNA interferes with cell function severely because it affects the cell cycle, induces cell apoptosis and leads to mutations^36^. 8-OHdG, a biomarker of oxidative damage to DNA, generated after repair of ROS-mediated DNA damage^37^. Significant association between 8-OHdG and the concentration of phthalate metabolites was observed in urine samples collected from 300 Brazilian children^38^. In our study, increased 8-OHdG and γ-H2AX levels were observed in vitro and in vivo exposed to DBP. These results indicated DNA damage was associated with DBP-induced toxicity.

As a key player of cell survival and DNA repair^39^, protective function of AKT activation has been confirmed in various cell-types and tissues, including vascular endothelial cells^40^, cortical neurons^24^, chondrocytes^41^ and so on. SC79 is a unique specific AKT activator that may be used to enhance AKT activity in various physiological and pathological conditions^24^. In the early stage of cerebral ischemia-reperfusion, SC79-induced AKT activation produced a decrease in the percentage of cortical infarct out of total cortical area^42^. In our studies, activation of AKT by SC79 also played a protective role in DBP-induced germ cells toxicity. However, SC79 did not reveal its protective role in a rat hearts ischemia and reperfusion model^43^, which indicated the role of SC79 is different depending on tissue specificity. Hence, more studies need to be conducted to explore the role of SC79 in different tissues.

PTEN is a phosphatase that is well-known for suppressing AKT-mTOR pathway through its lipid phosphatase activity. Our study indicates DBP suppressing AKT-mTOR pathway via increasing PTEN expression. The modification of DNA methylation provides a link between environmental exposures and gene expression. The most well-characterized epigenetic modification is DNA methylation, which is assumed to influence gene expression via forming 5-methylcytosine at CpG sites. The tumor suppressor PTEN is frequently regulated by aberrant promoter methylation as described above^44,45^. DNMTs are essential in the regulation of DNA methylation, but what triggers aberrant DNMTs expression is still unclear. Recently, DNMTs are predicted as miR-29 family (miR-29a, miR-29b, and miR-29c) targets^46^. miR-29b leads to a decrease in global DNA methylation by targeting DNMT3a and DNMT3b directly and DNMT1 indirectly^47^. In addition, HOTAIR downregulates miR-29b expression, leading to DNMT3b increase, which contributes to enhanced PTEN methylation^16^. To determine whether DBP induces PTEN expression via influencing its methylation status, we detected PTEN methylation and its transcriptional activity. Our results indicated DBP increased PTEN transcriptional activity via decreasing PTEN methylation. In addition, miR-29b was increased after treated with DBP and it played a critical role in DBP-induced toxicity according to bioinformatics analysis. DNMT3b is confirmed a target of miR-29b and miR-29b-mediated status of PTEN methylation is involved in the effects of DBP treatment.

Our previous results indicated DBP administration in pregnant mice could lead to its offsprings appearing decreased anogenital distance, body weight and testicular weight^6^. In addition, in pregnant mice exposed Di-isobutyl phthalate (DiBP) model, male offspring exposed either prenatally or postnatally were all revealed lower sperm concentration and motility compared with controls^48,49^. In the present animal experiment, we found DBP induced male mice offspring lower sperm concentration and motility is associated with AKT pathway and sperm flagellum-related genes. Sperm motility plays an important role in human reproduction. Men with multiple morphological abnormalities of the flagella (MMAF) have abnormal spermatozoa with severe flagellar defects, which impair sperm motility^50^. Previously, DNAH1, CCDC39, AKAP4, Cfap43, and Cfap44 had been confirmed to cause human MMAF^50^. Specifically, DNAH1 is required for the formation of the inner dynein arms, which is essential for normal sperm motility and its absence is associated with human fertility^51^. Cfap43 and Cfap44 knockout mice presented severe flagellar defects with complete infertility^52^. Additionally, CCDC39 also plays a vital role in assembly of inner dynein arms and is required for normal sperm motility in humans and dogs^53^. From the RNA sequencing results, we found many sperm flagellum-related genes, including DNAH1, CCDC39, AKAP4, Cfap43, and Cfap44, were downregulated in DBP administration group. qRT-PCR results were consistent with the RNA sequencing results. Due to the fact that AKT pathway is mainly regulated by phosphorylation and transcriptome sequencing cannot reveal phosphorylation level, we do not discuss AKT pathway in our sequencing results. These findings indicate DBP induce low sperm motility, which might be contributed by the decreased sperm flagellum-related genes and AKT pathway. In addition, AKT pathway could just be one of the signaling pathways involved in DBP-induced low sperm quality. Other signaling pathway should be explored in the future. Meanwhile, whether AKT pathway is associated with the expression of flagellum-related genes needs to be explored furtherly.

In conclusion, the present findings reveal DBP not only induces apoptosis/proliferation imbalance of germ cells, but also induces DNA damage in vitro and in vivo. DBP regulates PTEN/AKT pathway expression via decreasing PTEN methylation, which contributes to DBP-induced reproductive toxicity. Upregulation of miR-29b induced by DBP decreases DNMT3b expression, leading to PTEN demethylation. In addition, DBP reduces some major flagellum-related genes and AKT pathway expression, which is associated with DBP-induced low sperm motility. Collectively, these findings enrich the understanding of DBP in reproductive toxicity and more comprehensive studies remain to be conducted in the future.

## Materials and methods

### Cell culture

The mouse spermatogenesis pathway cell lines, GC-1 spg (catalog number: CRL-2053) and GC-2 spd (catalog number: CRL-2196) were obtained from American Type Culture Collection (ATCC, USA). GC-1 and GC-2 cells were cultured in Dulbecco’s modified Eagle’s medium with 10% fetal bovine serum (Gibco, USA) supplemented with 1% penicillin and streptomycin (Invitrogen, USA) at 5% CO_2_ and 37 °C incubator.

### Treatment of cells with chemicals

DBP purchased from Sigma-aldrich (catalog number: 524980) was dissolved in DMSO (<0.1% in the culture medium). GC-1 and GC-2 cells were treated with DBP in the concentration of 0, 2, 4, 6, 8, 10, 12, 14, 16 mg/L, respectively. SC79 (catalog number: S7863) were purchased from Selleck. Increasing concentrations of SC79 were pretreated in the GC-1 and GC-2 cells with for 2 h before the addition of DBP in the concentration of 10 mg/L. MK-2206 (catalog number: S1078) were purchased from Selleck. GC-1 and GC-2 cells were pretreated with MK-2206 at 3 μM according to manufacturer’s instructions (Selleck, USA).

### Ethics statement and animals

Adult male and female 8-week-old C57BL/6J mice were procured from the Center for Experimental Animals at Nanjing Medical University (Nanjing, China). Animals were housed at room temperature (23 ± 2 °C), relative 50 ± 10% humidity in an automatically controlled 12-hours light/dark cycle environment. Besides, these mice were supplied with abundant pellet chow and water during this experiment. After acclimation for one week, male and female rats were mated. Once female mouse pregnant, it would be housed individually in a conventional housing condition above. The day sperm was detected in the vaginal smear, which was considered gestational day 0.5 (GD 0.5). The study was carried out according to the Guide for the Care and Use of Laboratory Animals. All experimental protocols were approved by the Institutional Animal Care and Use Committee of Nanjing Medical University (approval number: IACUC-1807027).

### Animal treatment

DBP was suspended in corn oil (vehicle control, 99.5% pure, Solvent Factory, Shanghai, China) for gavage. Because corn oil does not have estrogen, it has been used as the vehicle for many toxicological studies^54,55^. DBP gavage dose and time-window selection was according to our previous study^6^. The pregnant dams were randomly assigned to 4 groups (*n* = 6) as follows: (1) Control group, pregnant dams were only given corn oil; (2) DBP group, these female mice were treated from GD 14.5 to GD 19.5 with 500 mg/kg/day of DBP in 1 mL/kg corn oil administered daily by oral gavage; (3) DBP + SC79 group, these pregnant dams were injected subcutaneously with 10 mg/kg/day SC79 while simultaneously receiving DBP (same as DBP group); (4) DBP + SC79 + MK-2206 group, these pregnant dams were injected subcutaneously with 5 mg/kg/day MK-2206 while simultaneously receiving DBP and SC79 (same as DBP + SC79 group). After delivery, all offsprings were allowed to grow with dams for 50 days. Then, testes of offsprings were carefully removed and one of testes was stored in 4% formaldehyde, and the other one was kept at −80 °C for subsequent biochemical measure. In addition, sperms were incubated in normal saline with 5% BSA for further analysis.

### Histological analysis

The chosen transverse sections from testicular tissues were performed histological analysis using hematoxylin and eosin (H&E) staining kit (catalog number: C0105, Beyotime, China) according to the manufacturer’s instructions. After stained, the testicular sections were examined under light microscopy (Olympus BX-51, Tokyo, Japan). Two experienced pathologists who were blinded to the experimental design evaluated testis structural changes and the number of seminiferous tubule/unit area of testes section. Five focuses from each mouse were randomly examined.

### Sperm motility analysis

Cauda epididymides were dissected and immersed into 2 mL of normal saline with 5% BSA and incubated for 5 min at 37 °C to allow sperm to release. The suspension was transferred to a hemocytometer and was allowed to stand for 5 min and then counted under a light microscope (Nikon, Japan) at ×200 magnification. In addition, sperm suspension was put into a Sperm Analysis Chamber and a computer assisted sperm analysis (CASA) system (Version 12 CEROS, Hamilton Thorne Research, Beverly, USA) was applied to detect the sperm motility. Two sperm motion parameters, motile percent and progressive percent, were evaluated in each sample.

### DNA methylation assessments

LUMA and BSP were applied to detect methylation status of global and PTEN promoter in germ cell lines and testicular tissues, respectively. The LUMA assay was performed as described in detail elsewhere^56^. PTEN CpG island was searched in UCSC Genome Browser. The bisulfite-sequencing analysis was carried out as described in our previous study^57^.

### Cell transfection

siRNA specifically targeting PTEN, AKT, or DNMT3b and scrambled oligonucleotides (si-NC) were purchased from Thermo Fisher Scientific. Transfection reagents used were Lipofectamine RNAiMAX (Thermo Fisher Scientific, USA). The pcDNA3.1-DNMT3b plasmid and the empty vector were synthesized by GenePharma (Shanghai, China). miR-29b-mimic, miR-29b-inhibitor and scrambled oligonucleotides (mir-29b-NC) were also synthesized by GenePharma. Transfection reagents used were Lipofectamine 3000 (Thermo Fisher Scientific, USA) following the manufacturer’s instructions. Sequences containing WT or mutant miR-29b-binding site in the 3′-UTR of DNMT3b were synthesized by GeneScript (Nanjing, China) and cloned into the luciferase expression plasmid pGL3-Basic (Promega, USA) to acquire the DNMT3b 3′-UTR reporter constructs (pGL3-WT-DNMT3b and pGL3-MUT-DNMT3b). Transfection reagents used were Lipofectamine 3000 (Thermo Fisher Scientific, USA) following the manufacturer’s instructions. The PTEN genomic DNA fragment −262 to +156 was amplified by Quantitative real-time PCR (qRT-PCR) and digested with KpnI and BglII and then inserted into promoter-loss luciferase expression plasmid pGL3-Basic (Promega, USA). The resulting plasmid was designated as pGL-262 to +156. Transfection reagents used were Lipofectamine 3000 (Thermo Fisher Scientific, USA) following the manufacturer’s instructions.

### Transient transfections and luciferase reporter assays

According to the manufacturer’s protocol, transient transfection of GC-1 and GC-2 cells was conducted using Lipofectamine™ 3000 (Invitrogen, USA). The cells were seeded into 96-well plates (1.5 × 10^4^/well) 24 h prior to transfection. Then, 100 ng luciferase reporter plasmids and 4 ng pRL-TK plasmid were transfected into cells using Lipofectamine™ 3000. After treated with DBP, 5-Aza-CdR, or M.SssI with 24 h, Luciferase assay was conducted by a Dual-luciferase Reporter Assay System (Promega, USA). Luciferase activity was normalized to the activity of pRL-TK. All results from at least three independent experiments.

### RNA sequencing

The total RNA was extracted from testicular tissues in a pubertal exposure to DBP rat model as previously described^58^ using an RNeasy Mini Kit (Qiagen) and on-column DNase digestion (RNase-Free DNase Set, Qiagen) to avoid contamination by genomic DNA. The cDNA library construction, sequencing, and transcriptome data analysis were performed by Personalbio Biotechnology Co., Ltd. (Shanghai, China). RNA-seq data used in this study have been deposited in the National Center for Biotechnology Information Sequence Red Archive (SRA) under the accession code SRR8528122-8528127.

### Quantitative real-time PCR (qRT-PCR)

Total RNA was extracted from cultured cells and testicular tissues using TRIzol reagent (Invitrogen, USA) and cDNA was synthesized using Primescript RT Reagent (Takara, Japan). The qRT-PCR was performed by using StepOne Plus Real-time PCR system (Applied Biosystems, USA) with SYBR^®^ Premix Ex Taq™ Reagent (Takara, Japan).

The primers used for qRT-PCR as follows:

PTEN-F: 5′-TGGATTCGACTTAGACTTGACCT-3′,

PTEN-R: 5′-GCGGTGTCATAATGTCTCTCAG-3′;

Cfap43-F: 5′-GATTCCCTAGTCAGAACGTCCA-3′,

Cfap43-R: 5′-CATGACGCCCACAATCCCAT-3′;

Cfap44-F: 5′-GATCGGGATCAAGGAATGAGGG-3′,

Cfap44-R: 5′-CCCCGTCGGTATATGAGTCAG-3′;

Dnah1-F: 5′-GGAGGTAGACAGTATCTGTGAGG-3′,

Dnah1-R: 5′-TGTTGGCAGCCCATTTGTCAT-3′;

Fsip2-F: 5′-TCTTCTCTCAACATGGAGCATGA-3′,

Fsip2-R: 5′-TGCCTATCTGCAATTTTGGACA-3′;

Ddx4-F: 5′-GAGAACACATCTACAACTGGTGG-3′,

Ddx4-R: 5′-CCTCGCTTGGAAAACCCTCT-3′;

Nme8-F: 5′-AGCTACAGTCAGTCGTCAATAGT-3′,

Nme8-R: 5′-AAACACGGGCTCACATTTATCT-3′;

Akap4-F: 5′-AGGACAACAAGATCAGGACCG-3′,

Akap4-R: 5′-CAGCAGCACCCTTGGAATC-3′;

Ldhc-F: 5′- TTGACGCTGATACGAACAAACT -3′,

Ldhc -R: 5′- AGACGATTTTTGGAGTGCTAAGG -3′;

Catsper2-F: 5′-GCTGATGCTATCCGTTCAAAGC -3′,

Catsper2-R: 5′-CCTGATCTCCTGACATGAGTTTC -3′;

Ccdc39-F: 5′-CAAGGGAGAGTGAGATCGAGA-3′,

Ccdc39-R: 5′-GTGAGCCGATTCTTCCAGCC-3′;

Eno4-F: 5′- CAGGCGATGGCGTACTACC -3′,

Eno4-R: 5′- CAGGTGCCCGTAGACATCC -3′;

Atp1a4-F: 5′-CTGGTGAGCTGAATCAGAAACC-3′,

Atp1a4-R: 5′-AAGACCCTTGGTCAAGTCCAC-3′;

GAPDH-F: 5′-AGGTCGGTGTGAACGGATTTG-3′,

GAPDH-R: 5′-GGGGTCGTTGATGGCAACA-3′.

### Cell counting kit-8 (CCK-8) proliferation assay

Cell viability was detected using CCK-8 proliferation assay (Dojindo, Japan). Cells were seeded at a density of 3 × 10^3^ cells/well in 96-well plates with 3 replicates. Cells were incubated at standard conditions for 1 h after 10 μl CCK-8 reagent was added and then the absorbance at 450 nm was measured with a microplate reader (Bio-Rad Laboratories, USA).

### EdU incorporation assay

The proliferation of GC-1 and GC-2 cells was detected using EdU Apollo®567 In Vitro Imaging Kit (Ribo Bio, China). Briefly, cells were seeded into 96-well plates at 4 × 10^3^ cells/well and incubated overnight. Prepare 50 μM 5-Ethynyl-2′- deoxyuridine (EdU) culture medium and then the prepared culture medium was added into 96-well plates at 100 μL/well incubated at standard conditions for 2 h. All procedures were performed according to the manufacturer′s protocol. Five fields of each well were randomly chosen and observed under fluorescence microscopy. All images were processed using Image J software and the proportion of EdU incorporated cells was calculated.

### TUNEL assay

Terminal deoxynucleotidyl transferase-mediated dUTP nick-end labeling (TUNEL) staining was performed to detect cell apoptosis levels. Briefly, cells were cultured in 6-well plates and exposed to DBP for 24 h, and then fixed in 4% paraformaldehyde followed by permeabilization with 0.2% Triton X-100. TUNE staining was performed according to the manufacturer’s instructions (Promega, USA) and observed under a fluorescence microscope (Nikon, Japan)

### Flow cytometry

Apoptosis assay was carried out using the APC Annexin V/PI apoptosis detection kit (BD Pharmingen, USA) according to the manufacturer’s protocol. Briefly, the cells were collected after treated with DBP for 24 h, and washed twice with cold PBS, and then resuspend cells in 1×Binding Buffer at a concentration of 1 × 10^6^ cells/ml. Sequentially, the cells were stained with APC Annexin V and PI at room temperature for 15 min in the dark and then analyzed by flow cytometry (Thermo Fisher, USA).

### Immunofluorescence

GC-1 and GC-2 cells were seeded in 6-well plates and were incubated overnight. After treated with DBP for 24 h, cells were fixed with 4% paraformaldehyde at 37 °C for 15 min, permeated with 0.1% Triton X-100 after washing with PBS three times and blocked by 2% bovine serum albumin (BSA) for 30 min. Next, the cells were incubated overnight at 4 °C with primary antibodies, including Ki67 (catalog number: ab15580, 1:1000 dilution, Abcam), 8-OHdG (catalog number: ab62623, 1:1000 dilution, Abcam), and γ-H2AX (catalog number: ab2893, 1:500 dilution, Abcam). 4′,6-Diamidine-2′-phenylindole dihydrochloride (DAPI) was used to stain the nuclei. Imaging was performed via fluorescence microscopy.

### Immunohistochemistry

After excising the testicular tissue, specimen was immediately placed into formalin, then fixed and paraffin-embedded. Testicular tissue sections were deparaffinized and rehydrated. Endogenous peroxidase activity was blocked in 3% H2O2 for 15 min. Tissue sections were incubated with primary antibodies Ki67 (catalog number: ab15580, 1:500 dilution, Abcam), 8-OHdG (catalog number: ab62623, 1:200 dilution, Abcam), and γ-H2AX (catalog number: ab2893, 1:200 dilution, Abcam) at 4 °C overnight and incubated with HRP conjugated secondary antibody for 1 h. All procedures were performed according to the manufacturer’s protocol. After stained by DAB, sections were observed under light microscopy.

### Western blot analysis

Total cellular proteins and mouse testicular tissues were extracted using the Total Protein Extraction Kit (Keygentec, China) and protein concentrations were measured using the BCA Protein Assay kit (Pierce, USA). Proteins were run on SDS-PAGE gels, blotted on nitrocellulose membrane, and immunodetected with primary antibodies against PTEN, PI3K, p-PI3K, AKT, p-AKT (Ser473), p-AKT (Thr308), mTOR, p-mTOR (S2448), cleaved caspase-3, Bax, Bcl-2, DNMT1, DNMT3a, DNMT3b (Cell Signaling Technology), and γ.H2AX (Abcam). β-actin (Abcam) were detected as controls. Signals were visualized by ECL blotting detection reagents (Thermo Fisher Scientific, USA) and exposed to X-ray films which were scanned and quantitatively analyzed using Image Lab Software (Bio-Rad, USA).

### Statistical analysis

All data are represented by mean ± standard deviation (SD) from three independent experiments. Statistical analysis was performed using variance tests (two-way ANOVA and one-way ANOVA). Data sets were compared using two tailed, unpaired *t*-test. *p < 0.05 was considered as the significance level; the data were marked as *p < 0.05, **p < 0.01, and ***p < 0.001.

## Supplementary information

Legends

Figure S1

Figure S2
