# Supplementary material for: Di-n-butyl phthalate epigenetically induces reproductive toxicity via the PTEN/AKT pathway
Source: Cell Death Dis. 2019 Apr 5;10(4):307. doi: 10.1038/s41419-019-1547-8 (PMC6450951; doi:10.1038/s41419-019-1547-8)
Supplement: Supplementary file 1 — Legends [file 41419_2019_1547_MOESM1_ESM.docx]

**Supplementary Material**

**Figure S1. SC79 attenuated DBP-induced decreased cell viability of GC-1 and GC-2 cells.** (A and B) Cell viability was detected by CCK-8 proliferation assay in GC-1 (A) and GC-2 (B) cells after pretreatment of the cells with increasing concentrations of SC79 for 2h before the addition of DBP in the concentration of 10 mg/L. (C) qRT-PCR was used to detect the efficiency of AKT knockdown in GC-1 and GC-2 cells. (D) Representative WB images showing the efficiency of AKT knockdown in GC-1 and GC-2 cells. (E) Representative WB images showing the effect of SC79 on p-AKT and AKT expression. (F) Representative WB images showing the effect of MK-2206 on p-AKT and AKT expression. (G and H) Cell viability was detected by CCK-8 proliferation assay in GC-1 (G) and GC-2 (H) cells after DBP treatment, SC79 treatment, AKT knockdown or MK-2206 treatment. All measurements are shown as the means ± SD from three independent experiments, **p < 0.01, ***p < 0.001.

**Figure S2. Differentially expressed miRNAs and miRNA-Gene-Network.** (A) Hierarchical cluster analysis of differentially expressed (DE) miRNAs were acquired in rat testes triggered by DBP treatment compared with the control groups. (B) Box nodes represent miRNAs, and cycle nodes represent the predicted target genes. Edges show the inhibitory effect of miRNA to its predicted targets. Degree means the contribution one miRNA to the genes around or the contribution one gene to the miRNAs around. The key miRNAs and genes in the network always have the highest degrees.
