# Supplementary figures and images for: Di-n-butyl phthalate epigenetically induces reproductive toxicity via the PTEN/AKT pathway
Source: Cell Death Dis. 2019 Apr 5;10(4):307. doi: 10.1038/s41419-019-1547-8 (PMC6450951; doi:10.1038/s41419-019-1547-8)

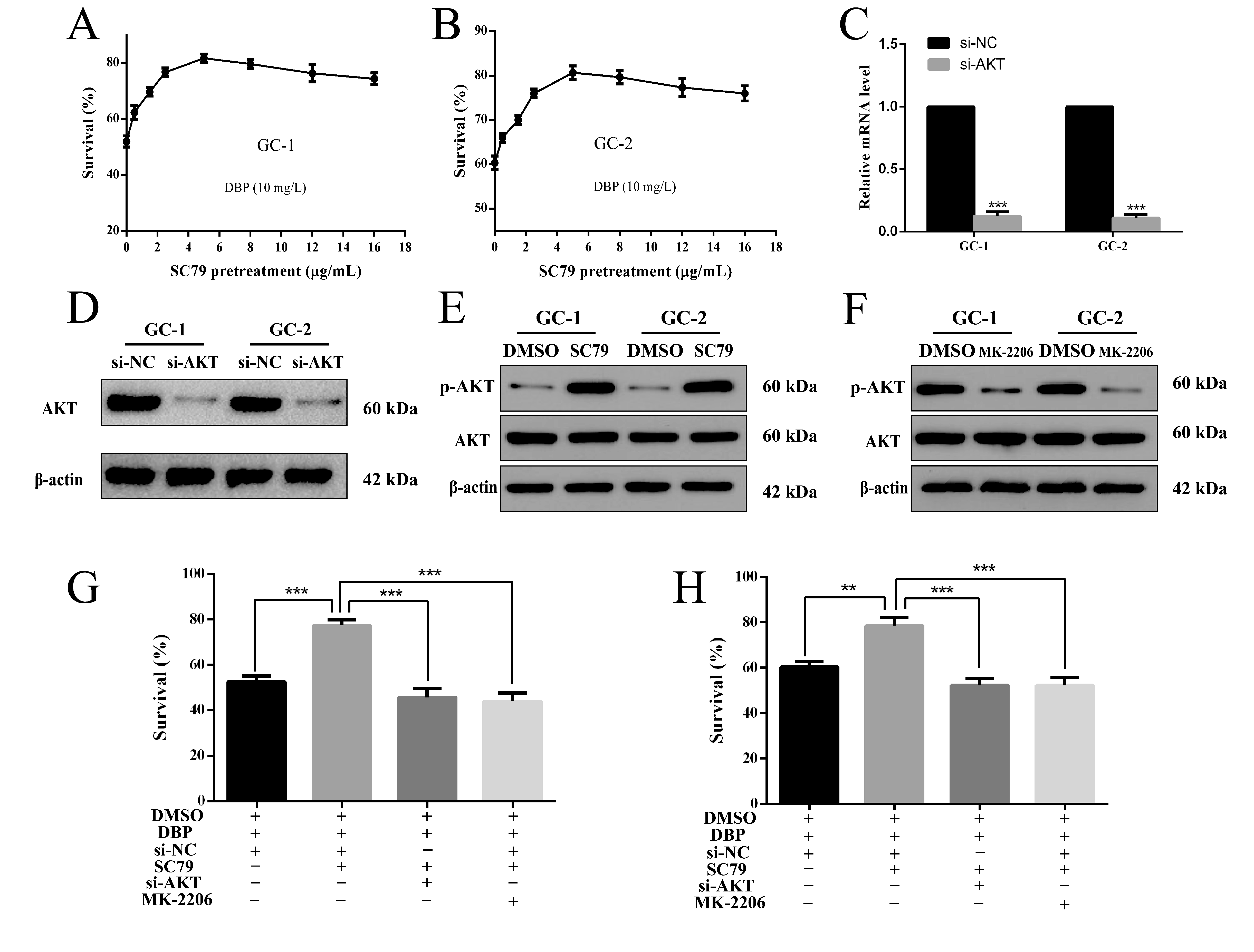

Supplement: Supplementary file 2 — Figure S1 [file 41419_2019_1547_MOESM2_ESM.tif]

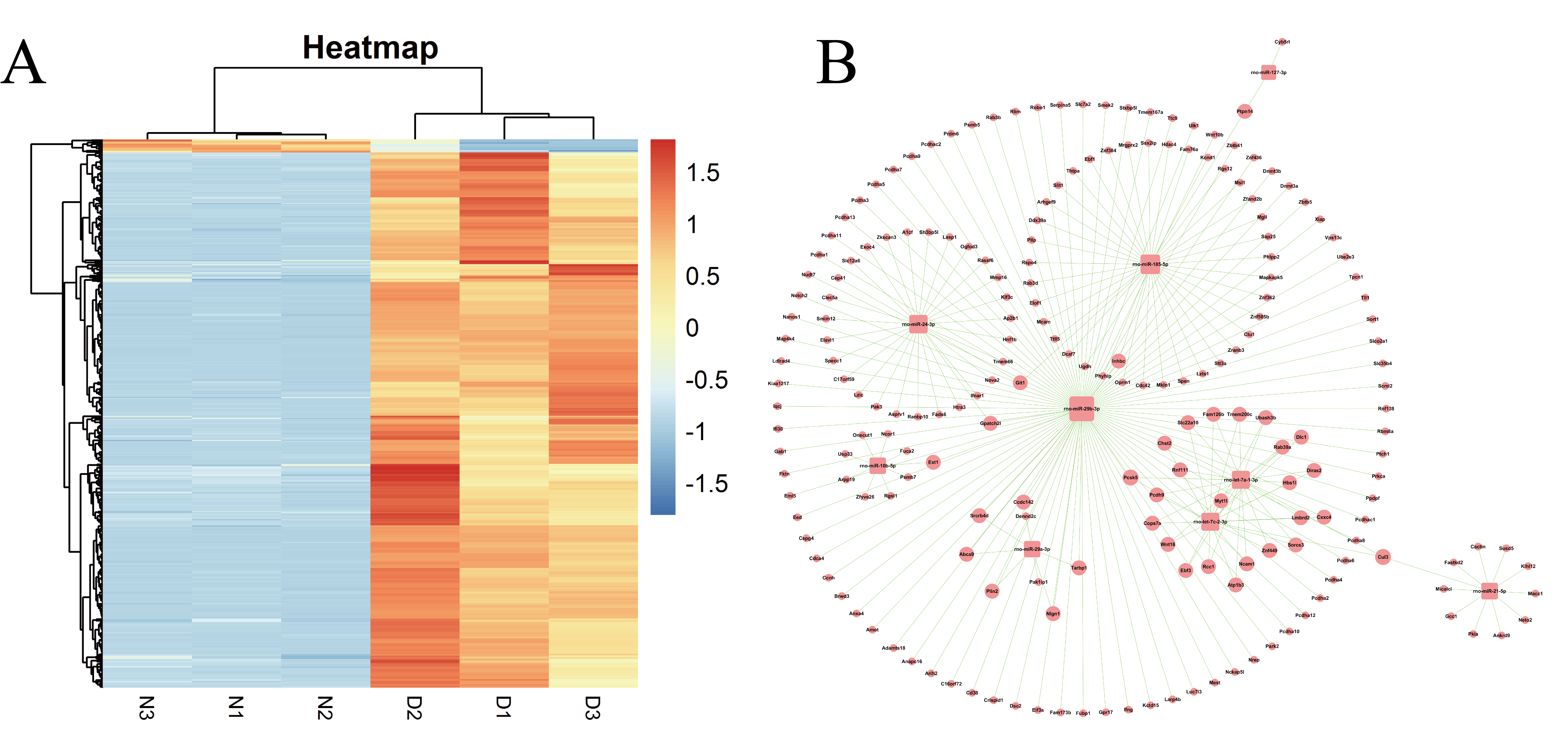

Supplement: Supplementary file 3 — Figure S2 [file 41419_2019_1547_MOESM3_ESM.tif]
